# Statins attenuate cholesterol-induced ROS via inhibiting NOX2/NOX4 and mitochondrial pathway in collecting ducts of the kidney

**DOI:** 10.1186/s12882-022-02815-6

**Published:** 2022-05-13

**Authors:** Ani Wang, Yu Lin, Baien Liang, Xiaoduo Zhao, Miaojuan Qiu, Hui Huang, Chunling Li, Weidong Wang, Yonglun Kong

**Affiliations:** 1 Cardiovascular Center, The 5thAffiliated Hospital, Sun Yat-Sen University, Zhuhai, 519000 China; 2 Department of Pathology, Zhujiang Hospitial, Southern Medical University, Guangzhou, 510282 China; 3 Department of Pathophysiology, Zhongshan School of Medicine, Sun Yat-Sen University, 74# Zhongshan 2nd Road, Guangzhou, 510080 China; 4 Institute of Hypertension, Zhongshan School of Medicine, Sun Yat-Sen University, Guangzhou, 510080 China; 5 Research Center, The 7th Affiliated Hospital, Sun Yat-Sen University, Shenzhen, 518107 China; 6 Department of Cardiology, The 8th Affiliated Hospital, Sun Yat-Sen University, Shenzhen, 518033 China

**Keywords:** Cholesterol, Statins, ROS, Collecting ducts

## Abstract

**Background:**

Statins therapy has been primarily recommended for the prevention of cardiovascular risk in patients with chronic kidney diseases. Statins has also been proved some benefits in lipid-induced kidney diseases. The current study aims to investigate the protection and underlying mechanisms of statins on renal tubular injuries induced by cholesterol overloaded.

**Methods:**

We used tubular suspensions of inner medullary collecting duct (IMCD) cells from rat kidneys and mouse collecting duct cell line mpkCCD cells to investigate the effect of statins on reactive oxygen species (ROS) production induced by cholesterol. Protein and mRNA expression of NADPH oxidase 2 (NOX2) /NOX4 was examined by Western blot and RT-PCR in vitro studies and in rats with 5/6 nephrectomy and high-fat diet. Mitochondrial morphology and membrane potential was observed by Mito-tracker and JC-1.

**Results:**

Statins treatment was associated with decreased NOX2 and NOX4 protein expression and mRNA levels in 5/6Nx rats with high-fat diet. Statins treatment markedly reduced the ROS production in IMCD suspensions and mpkCCD cells. Also, statins reduced NOX2 and NOX4 protein expression and mRNA levels in cholesterol overload mpkCCD cells and improved mitochondrial morphology and function.

**Conclusion:**

Statins prevented ROS production induced by cholesterol in the kidney, likely through inhibiting NOXs protein expression and improving mitochondrial function. Statins may be a therapeutic option in treating obesity-associated kidney diseases.

**Supplementary Information:**

The online version contains supplementary material available at 10.1186/s12882-022-02815-6.

## Introduction

Hyperlipidemia is one of the most prevalent diseases in both developing and developed countries. Hyperlipidemia has been hypothesized to play an important role in the progression of kidney injury [1], at least partially, due to deleterious renal lipid accumulation. Lipids may cause both glomerular and tubular cell injury and promote renal disease progression [2]. Several molecular mechanisms mediating cellular dysfunction and injury caused by lipid accumulation in renal tubular segments have been investigated, including the generation of reactive oxygen species (ROS), damages of multiple organelles, release of proinflammatory and profibrotic factors, and lipid-induced apoptosis [3]. Our recent study demonstrated that kidney collecting duct cell injuries induced by lipid accumulation may cause retention of water, presumably leading to an increase in preload and blood pressure [4].

In physiological condition, ROS plays an important role in proliferation, differentiation, and apoptosis of various cells, including renal collecting duct cells [5]. Oxidative stress during various metabolic disturbances (e.g. hyperlipidemia) may cause damages of kidney epithelial cells by affecting several signaling pathways, leading to end stage renal disease (ESRD) [6]. Generally, ROS generation is mediated by two pathways, enzymatic and nonenzymatic pathways. One of critical enzymatic pathways is nicotinamide adenine dinucleotide phosphate oxidase (NADPH oxidase, NOX) composed of two isoforms NOX4 and NOX2, NOX4 is highly expressed in kidney tubular epithelial cells which constitutively produce hydrogen peroxide (H_2_O_2_), a prevalent ROS detected [7]. As the major NADPH isoform in the kidney, NOX4 contributes to redox processes in diabetic and obese kidneys [8]. Conversely, mitochondrial electron transport chain (mETC) deficiencies, advanced glycation end products (AGEs), glucose auto-oxidation, etc. were identified as nonenzymatic pathways [9].

Renal oxidative stress is often a consequence of pro-oxidant enzyme-induced ROS production and concomitant depletion of antioxidants. Among the enzymatic systems implicated in ROS generation in the kidney, the NOXs appear to be the key contributors. Besides, mitochondrial dysfunction (MtD) is also involved in cholesterol- induced kidney damage [10], since intracellular levels of ROS can be induced by the electron leakage from mitochondrial respiratory chain. Cholesterol in excess can cause injuries in glomerular, tubular and tubulointerstitial cells through multiple mechanisms, one of which is increased production of ROS through both mitochondria and NADPH oxidases pathways.

HMG-CoA reductase inhibitors statins clearly reduce the risk of cardiovascular disease and mortality; they attenuated proteinuria and preserved renal function independent of other variables. Clinical evidence has shown that statins treatment is beneficial to the kidney of patients with hyperlipidemia [11]. HMG-CoA reductase was found expressed in collecting ducts and proximal tubular epithelial cells, which was greatly increased by high-fat diet, indicating a potential role of tubular epithelial cells in cholesterol regulation in the kidney. Statins may inhibit NADPH oxidase stability on the plasma membrane [12] and protect vascular damage in diabetic patients by their antioxidant properties [13]. Our recent data demonstrated that hyperlipidemia induced NLRP3 activation and exacerbated kidney injury in animals with high-fat diet, which was at least partially prevented by statins treatment [4]. NLRP3 inflammasome may sense directly the presence of ROS produced by damaged mitochondria [5] and assembly could be triggered by extracellular ATP and ROS [14]. In our study, the protective effect of statins is at least partially attributed to inhibition of NLRP3 components [4], however, whether statins prevent lipid-induced kidney injuries through inhibiting ROS was not investigated.

The purpose of the present study is to examine whether statins prevents ROS production induced by cholesterol and potential molecular mechanisms in rats with chronic kidney disease and in cultured collecting duct cells.

## Materials and methods

### Animals

All animal procedures were approved by the Animal Care and Use Committee of Sun Yat-sen University (Ethics Committee of ZSSOM on laboratory Animal Care No. 2016–048; Guangzhou, China). Animal experiments are described previously [4]. Briefly, on the day of the operation, right unilateral nephrectomy was performed in Male Sprague–Dawley rats and renal mass reduction was obtained by ablation of two-thirds mass of the left kidney one week later. For the sham-operation rats a laparotomy was performed, and the renal pedicle manipulated without any removal of renal mass. Rats were divided into three groups: 5/6Nx treated with standard laboratory chow; 5/6Nx rats treated with 60% cholesterol chow; and 5/6Nx rats treated with 60% cholesterol chow and atorvastatin (20 mg/kg BW/day). Rats were sacrificed 12 weeks after 5/6Nx. The animals were housed in a room with 12-h light/dark cycle with a temperature of 25℃.

### Histologic analysis

Histologic experiments are described previously [4]. Paraffin-embedded kidney sections used for IHC studies were dewaxed, rehydrated, and incubated with primary antibodies against NOX2 (Santa Cruz Biotechnology, USA, 1:400) and NOX4 (Santa Cruz Biotechnology, USA, 1:400) overnight at 4℃. The sections were subsequently incubated with secondary antibodies, treated with diaminobenzidine, counterstained with hematoxylin and examined as previously reported. NOX2 and NOX4 mean density (IOD SUM/area) was analyzed by Image J software.

### Inner medullary collecting duct (IMCD) suspension preparation and treatments

Rat IMCD suspensions were prepared as previously described [4]. Primary IMCD cells were pretreated with or without atorvastatin (20 μM) for 30 min, and then incubated with cholesterol (C4591, Sigma, USA, 200 ng/mL) or a vehicle for 6 h.

### mpkCCD preparation and treatments

mpkCCD cell culture are described previously [4]. To examine the effects of simvastatin on cholesterol induced inflammation activation, immortalized mouse cortical collecting duct cell line (mpkCCD cells) were seeded on 6 wells plates and then incubated in serum-free medium for 12 h before cholesterol and simvastatin treatment. Cholesterol treatment was last for 24 h. To examine the effects of simvastatin on cell viability, mpkCCD cells were seeded into 96-well plates at a density of 5000 cells per well. After 24 h simvastatin treatment, the cell viability was determined by using the Cell Counting Kit-8 according to the manufacturer’s instructions (Beyotime, Shanghai, China).

### Western blotting

Methods of Western blotting is described previously [4]. The mpkCCD cells were lysed in protein lysis buffer for 15 min on ice before protein was extracted. Immunoblotting was performed with primary antibodies against NOX2 (Santa Cruz Biotechnology, USA, 1:1000), NOX4 (Santa Cruz Biotechnology, USA, 1:1000), Cleaved-caspase3 1:1000 (Cell signaling technology, USA, 1:1000) followed by the addition of horseradish peroxidase-labelled secondary antibodies. The blots were visualized with ECL detection systems. Densitometric analysis was performed using AlphaEase Software. The experiment was repeated three times. The blots were cut prior to hybridization with antibodies during blotting, all blots with membrane edges visible are provided in supplementary figures.

### Quantitative RT-PCR

The protocol of quantitative RT-PCR is described [15]. Total RNA was extracted from cultured mpkCCD cells and inner medullar of rats according to the manufacturer’s instructions for Trizol reagent (Invitrogen). Total RNA (1,000 ng) was used for reverse transcription using PrimeScript RT Reagent Kit Perfect Real Time Kit (Takara Bio). The cDNA was used for quantitative real-time PCR analysis (qPCR) using SYBR Premix Ex Taq (Perfect Real Time) (Takara Bio). All samples were analyzed in triplicate. The calibrator sample was selected from PBS-treated tissue or cell samples, and GAPDH was used as an internal control. Relative amounts of mRNA were normalized by GAPDH and a control sample and calculated by using the comparative Ct (2 − ΔΔCT) (cycle threshold) method. Signals from the control group were assigned a relative value of 1.0. Primers were designed based on previous publications or on the primer bank, and the sequencing of primers are provided (Tables 1 and 2).

**Table 1:** Primer sequences for RT-PCR (Rat)

Target Gene	Primer Sequence
NOX2 F	CTTTAGCATCCATATCCGCATT
NOX2 R	GACTGGTGGCATTGTCACAATA
NOX4 F	GAGCAACAAACCTGTCACCAT
NOX4 R	TGCTGATACACTGGGACAATG
NOS2 F	CTGCATGGAACAGTATAAGGCAAAC
NOS2 R	CAGACAGTTTCTGGTCGATGTCATGA
NOS3 F	ACGTGGAGATCACCGAGCTC
NOS3 R	GTGCTCATGTACCAGCCACTG

**Table 2:** Primer sequences for RT-PCR (mouse)

Target Gene	Primer Sequence
NOX2 F	TGGCTCCACTGGGAATTGC
NOX2 R	CAAACCCGGCATCATGGGA
NOX4 F	GAAGGGGTTAAACACCTCTGC
NOX4 R	ATGCTCTGCTTAAACACAATCCT
NOS2 F	CAGGGAGAACAGTACATGAACAC
NOS2 R	TTGGATACACTGCTACAGGGA
NOS3 F	GTCTGGAGGGCTAAGCAGTC
NOS3 R	GCAAGGAAGGTTGACAGTATGC

### ROS production studies

To determine the intracellular ROS production, cells were incubated with 5 μM DCFH-DA probe for 30 min at 37℃ before subjected to immunofluorescence analysis or flow cytometry analysis.

### Immunofluorescence

The protocol of immunofluorescence staining is described previously [16]. mpkCCD cells cultured on 35 mm glass were subjected to the confocal analysis after fixed in 4% paraformaldehyde and permeabilized with 0.5% Triton X-100 for 15 min at room temperature. After being blocked with 10% goat serum in TBST at room temperature for 1 h, cells were incubated with NOX2 and NOX4 antibodies overnight at 4℃. Cells were washed and then incubated with Alexa Fluor-555-conjugated goat anti-rabbit secondary antibody for 1 h at room temperature. The nuclei were counterstained in blue with 4, 6-diamidino-2-phenylindole (DAPI).

### Mitochondrial staining and membrane potential measurement

The experimental steps were described previously [16]. A total of 100 nM Mito-tracker green solution was added to cells on a glass slide. The mpkCCD cells were incubated for 30 min at 37℃ to stain the mitochondrial membrane. The staining solution was removed, and cells were observed using microscopy. 5,5’,6,6’-tetrachloro-1,1’,3,3’-tetraethylbenzimidazolylcarbocyanine iodide (JC-1) (Med Chem Express, Shanghai, China) staining method was used to detect the mitochondrial membrane potential. Briefly, cells were washed with PBS buffer and then incubated with JC-1 fluorescent dyes for 30 min at 37℃. The cells were washed with PBS buffer and applied to microscopy analysis.

### Statistical analysis

Results are presented as the means ± SEM. Data were analyzed by one-way ANOVA and Student-Newman–Keuls tests for multiple comparisons. Statistical significance was accepted at the *p* < 0.05 level.

## Results

### Atorvastatin treatment reduced high-fat diet induced high expression of NOX2 and NOX4 in collecting duct of kidney

**Fig. 1:**
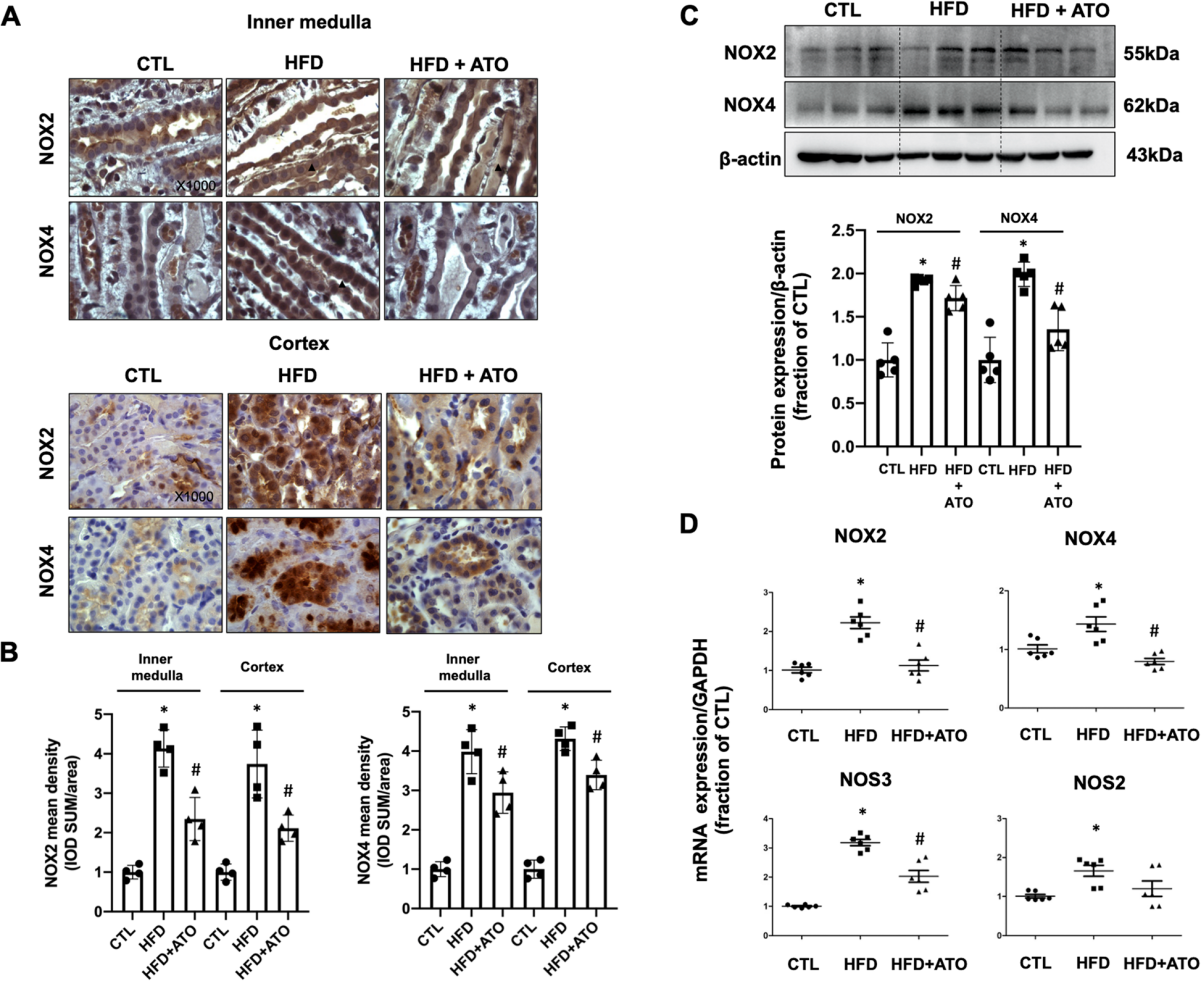
**A** Immunohistochemistry staining of NAPDH oxidase NOX2 and NOX4 in kidney sections of 5/6Nx and high-fat diet rats with or without atorvastatin treatment. **B** Quantitative analysis for NOX2 and NOX4 staining in inner medulla and cortex of 5/6Nx and high-fat diet rats with or without atorvastatin treatment. **C** Protein abundance of NOX2 and NOX4 were detected by western blotting and corresponding semiquantitative densitometry analysis in kidney of 5/6Nx and high-fat diet rats with or without atorvastatin treatment. **D** mRNA level of NOX2, NOX4, NOS3 and NOS2 in 5/6Nx and high-fat diet rats with or without atorvastatin treatment. Original magnification, X1000. Scale bars, 10 μm

Cholesterol accumulation in tubular epithelial cells may induce ROS production and lead to tubular damages. NOX2 and NOX4 are critically involved in ROS production in the kidney. In the kidney collecting duct epithelial cells, NOX2 and NOX4 protein expression are mainly seen in both apical membrane and cytoplasma. 5/6Nx and high-fat diet for 12 weeks notably increased the protein expression of NOX2 and NOX4 in the kidney, which was clearly prevented by atorvastatin treatment (Fig. 1A and B). As shown in Fig. 1C, the protein abundance levels of NOX2 and NOX4 were found to be increased in the kidneys of 5/6Nx rats fed with high-fat diet. Treatment with atorvastatin down regulated the protein expression of NOX2 and NOX4. Renal mRNA levels of NAPDH oxidase markers (NOX2, NOX4, NOS3, NOS2) were detected in 5/6Nx rats with high-fat diet. 5/6Nx and high-fat diet was associated with significantly increased mRNA levels of NOX2, NOX4, NOS3, and NOS2; again, atorvastatin markedly decreased their mRNA levels (Fig. 1D).

### Simvastatin treatment reduced ROS production induced by cholesterol in mpkCCD cells

**Fig. 2:**
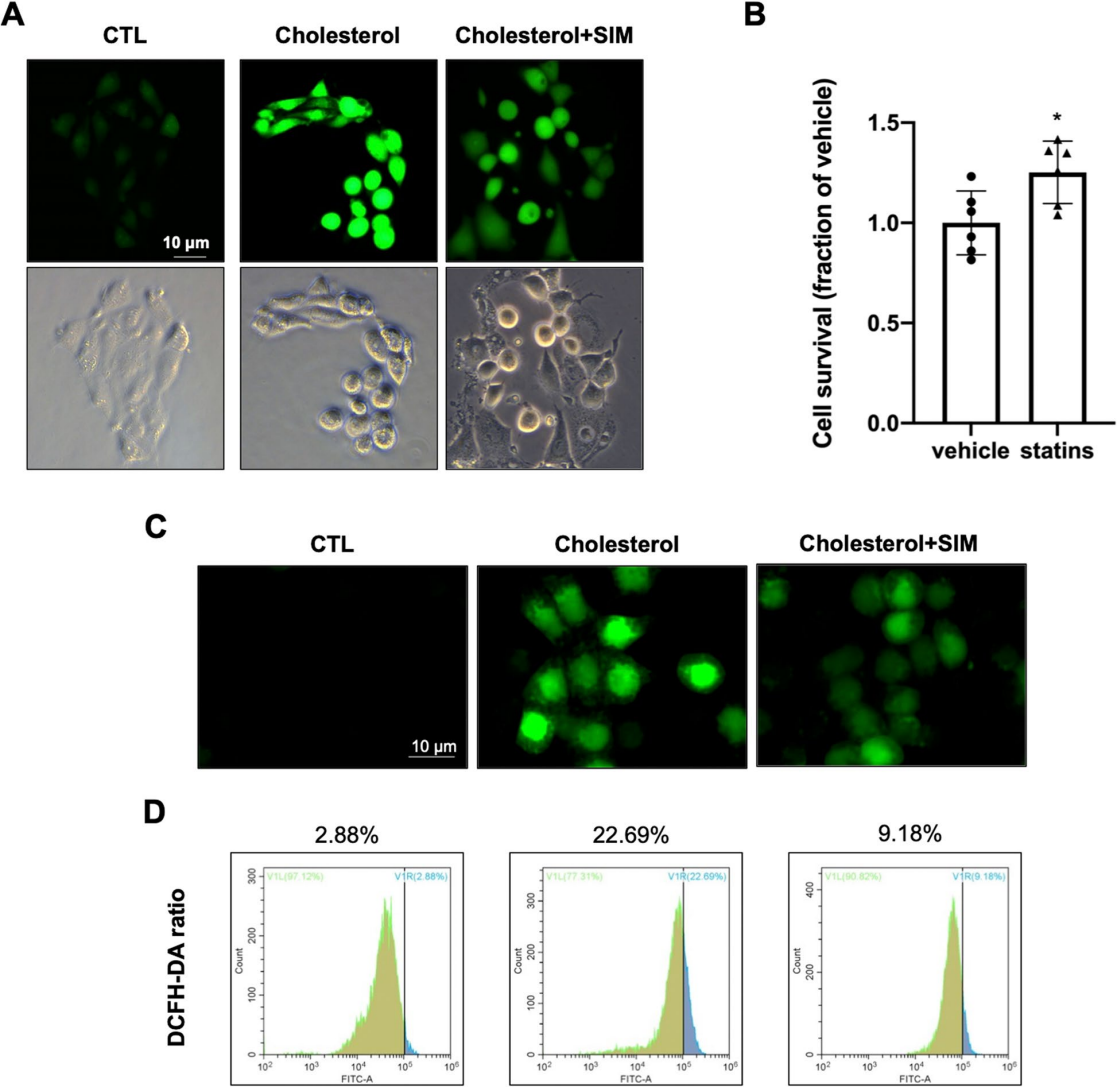
**A** ROS production was detected in cholesterol overload primary IMCD suspensions with or without simvastatin. **B** Cell survival ratio of mpkCCD cells treated with simvastatin for 24 h assessed by CCK. **C** Immunofluorescence images showed that simvastatin reduced cholesterol induced intracellular ROS production in mpkCCD cells. **D** ROS production in cholesterol-treated mpkCCD cells with or without simvastatin were quantified by flow cytometry

We established an in vitro IMCD cell cholesterol overload model to further examine a protective role of statins. Cholesterol treatment for 24 h markedly caused ROS production in IMCD suspension as seen by immunofluorescence, which was prevented by simvastatin (Fig. 2A). Cell survival ratio showed that simvastatin treatment slightly increased cell viability in mpkCCD cells (Fig. 2B). The effect of statins on ROS production induced by cholesterol was further examined in mpkCCD cells. ROS production was detected by DCFH-DA probe. Immunofluorescence demonstrated that DCFH-DA staining was markedly increased in cholesterol overload group compared to controls. While statins reversed marked increase of DCFH-DA staining induced by cholesterol (Fig. 2C). Flow-cytometry analysis confirmed increased ROS production after cholesterol treatment, which was notably reduced by simvastatin from 22.69% DCFH-DA positive ratio in cholesterol overloaded group to 9.18% (Fig. 2D).

### Simvastatin treatment prevented upregulation of NOX2 and NOX4 protein expression in mpkCCD cells treated with cholesterol

**Fig. 3:**
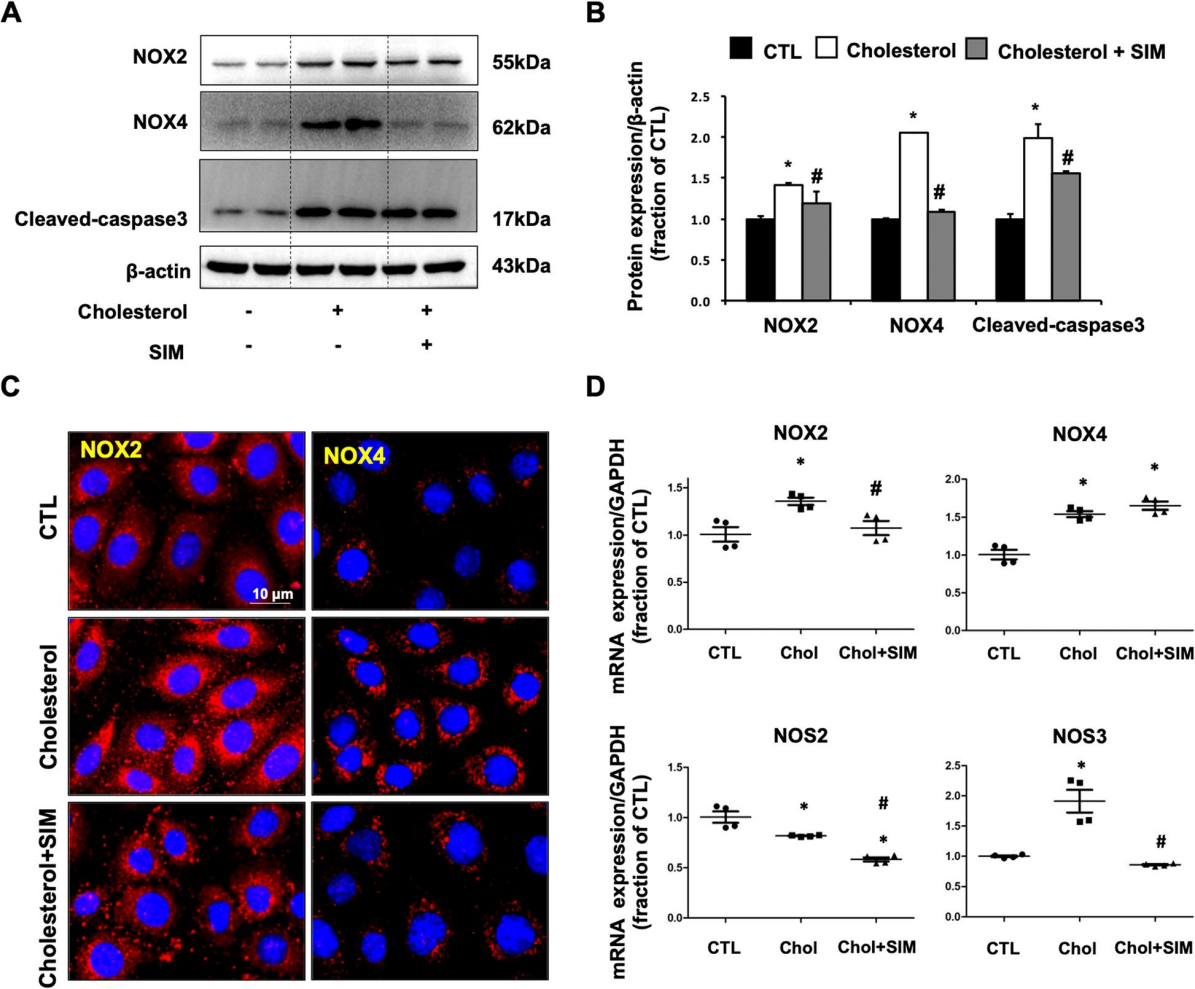
**A** and **B** Protein abundance of NOX2, NOX4, and Cleaved-caspase3 were detected by western blotting and corresponding semiquantitative densitometry analysis in cholesterol-treated mpkCCD cells with or without simvastatin treatment. **C** Immunofluorescence of NOX2 and NOX4 in cholesterol-treated mpkCCD cells with or without simvastatin treatment. **D** mRNA level of NOX2, NOX4, NOS2, and NOS3 in cholesterol-treated mpkCCD cells with or without simvastatin treatment. *P < 0.05 compared with CTL. ^#^*P* < 0.05 compared with Cholesterol. Scale bars, 10 μm

In mpkCCD cells, cholesterol treatment for 24 h induced upregulated protein expression of NOX2 and NOX4, consistent with increased ROS production. Cleaved-caspase3 protein expression was also increased in cholesterol treatment group, indicating cell damage in response to cholesterol overloaded. Simvastatin treatment markedly decreased protein abundance of NOX2 and NOX4 and attenuated an increase of Cleaved-caspase3 expression induced by cholesterol (Fig. 3A and B). Immunofluorescence showed that cholesterol treatment caused markedly increased staining density of NOX2 (red in left panel) and NOX4 (red in right panel) around nuclei in mpkCCD cells, while simvastatin treatment prevented the expression of NOX2 and NOX4 dramatically (Fig. 3C). NOX2 and NOX4 mRNA levels were significantly increased in cholesterol treated mpkCCD cells, simvastatin treatment reduced mRNA levels of NOX2, but not NOX4. NOS2 and NOS3, two molecules downstream of NOX2 and NOX4 signaling pathway, are involved in ROS production. Interestingly, mRNA level of NOS3 was upregulated by cholesterol treatment which can be abolished by simvastatin treatment, whereas NOS2 mRNA level was downregulated by cholesterol treatment and simvastatin treatment further decreased NOS2 mRNA level during cholesterol treatment (Fig. 3D).

### Simvastatin treatment protected mitochondria dysfunction in mpkCCD cells treated with cholesterol

**Fig. 4:**
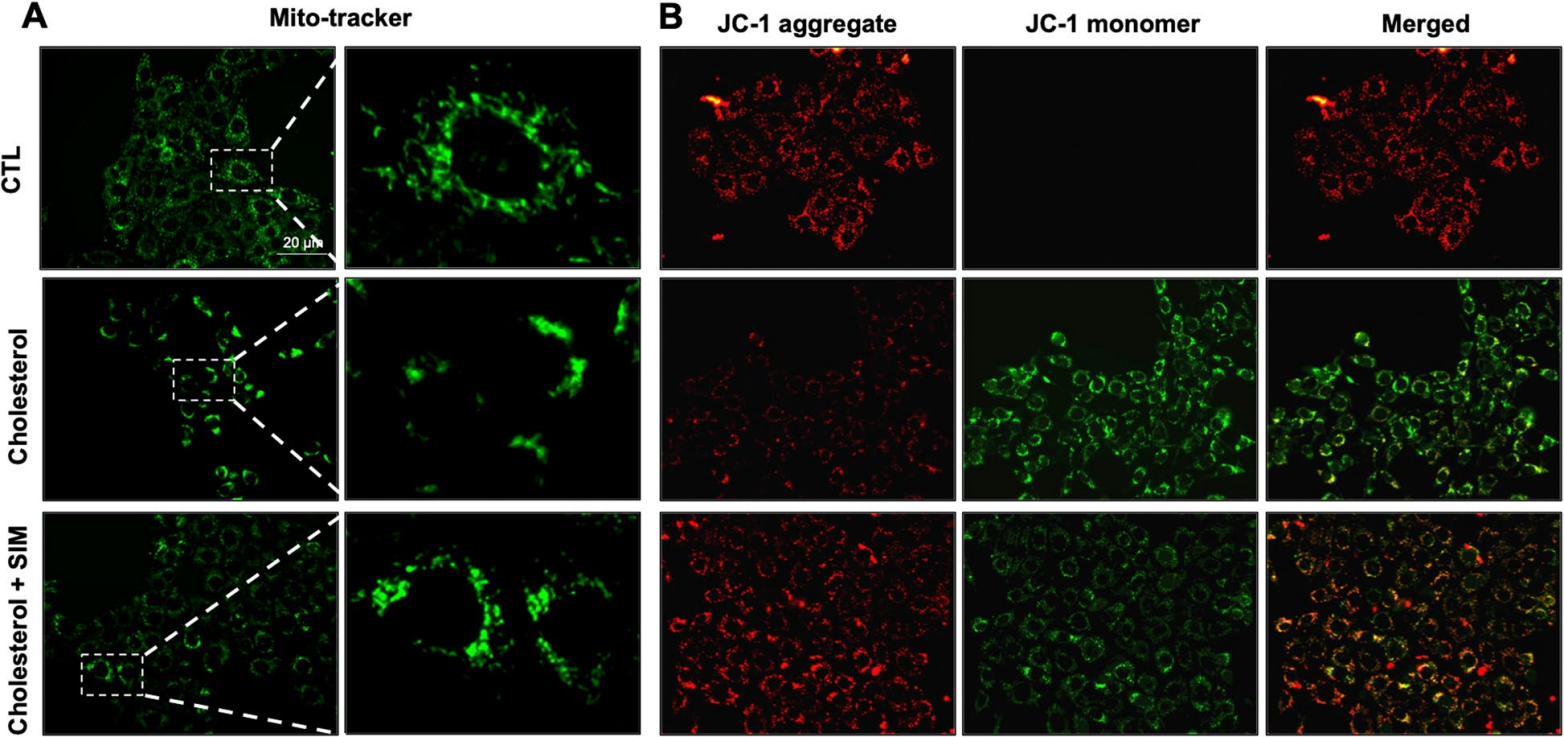
**A** Confocal microscopy images of Mito-tracker (green) in mpkCCD cells with or without simvastatin treatment for 24 h. **B** Representative images of JC-1 staining showing JC-1 aggregate (red) and monomer (green) in mpkCCD cells with or without simvastatin treatment for 24 h. Scale bars, 20 μm

Beside NAPDH oxidase, mitochondrial dysfunction (MtD) is another source of ROS production in damaged kidney. To determine whether simvastatin protected cholesterol-induced mitochondrial dysfunction, we examined mitochondrial status and membrane potential in cholesterol-treated mpkCCD cells with pretreatment of simvastatin. By staining with a mitochondrial probe Mito-Tracker in mpkCCD cells, it was demonstrated that more damaged mitochondria accumulated around nuclei with cholesterol treatment, while simvastatin treatment reversed the totally intensity and reduced mitochondrial fragmentation/fission induced by cholesterol (Fig. 4A). To further examine the protective effect of simvastatin, mitochondrial membrane potential was measured by using JC-1 in mpkCCD cells treated with cholesterol. JC-1 exists as either a monomer (green-fluorescent) at depolarizing mitochondria or an aggregate (red-fluorescent) at polarizing mitochondria. In normal mitochondrial membrane potential, mitochondria were marked by red fluorescence of JC-1 with no green fluorescent. Cholesterol treatment led to downregulation of red JC-1 signals together with diffused green monomer fluorescence signals (Fig. 4B). The switch from aggregate to monomer JC-1 signal indicated the loss of mitochondrial membrane potential in these cholesterol-treated cells, which at least partially reversed by simvastatin (Fig. 4B).

Taken together, our data suggest that increased NOX2 and NOX4 protein expression and mitochondrial dysfunction induced by cholesterol lead to over production of ROS, leading to apoptosis and cell death in the kidney. Statins treatment protected tubular cells from cholesterol-induced damage by downregulating NOX2/NOX4 protein expression and improving mitochondrial function.

## Discussion

In the current study, we found an anti-oxidative stress effect of statins in the kidney. Statins downregulated protein and mRNA expression of NOX2/NOX4 and improved mitochondrial function, by which statins suppressed ROS production and prevented cholesterol-induced kidney injuries. The present data supported our previous findings [4] that statins may protect kidney injury from cholesterol overload, independent of its property of lowering lipid.

Consequent ROS production can trigger abnormal signaling pathways involving diverse signaling mediators such as transcription factors, inflammatory cytokines, chemokines, and vasoactive substances [17]. Persistently, increased expression and activation of these signaling molecules contribute to the functional and structural changes in the kidney. ROS-induced kidney injuries have been recognized in diabetic and obese nephropathy. High glucose induced mitochondrial damage in renal tubular cells which was associated with ROS generation. ROS, acting as a key messenger in the signaling transduction, is involved in obese-associated kidney fibrosis [18] and in diabetic nephropathy [19]. It has been previously reported that fatty acid modulates mitochondrial ROS production by several mechanisms, including interactions among components of the respiratory chain, there by inhibiting the electron transport [20]. Nitric oxide (NO) is produced from the conversion of L-arginnine by NO synthase (NOS) and mediated a variety of biology processes such as ROS production. A recent study demonstrated that cholesterol downregulated NOS2 gene level and protein expression in kidneys of FVB/N mice fed with 1% cholesterol diet for 6–8 weeks [21]. Consistent with this, we showed HFD increased NOS2 mRNA level in 5/6Nx rats with high-fat diet for 12 weeks. These data suggested that cholesterol may mediating ROS production in different stages of chronic kidney diseases. In vitro results shown in Fig. 3D demostrated that Atoravstatin treatment have no effect on mRNA level of NOX4 but decreased it’s protein expression dramatically (Fig. 3A). The underlying mechanism is still unknown, but it may be associated with post translational modifications of NOX4 and NOS2 induced by statins [22]. Our finding showed that cholesterol also increased ROS production which can be mediated by NAPDH oxidase and mitochondrial damage in the kidney. These data suggests that during dyslipidemia both fatty acid and cholesterol trigger ROS production by mitochondrial and enzymatic pathways, leading to kidney injuries.

Evidence has shown that accumulation of lipid droplets in proximal tubular epithelial cells could be one of the causes to induce ROS overproduction [23]. Although a protective role of statins by lipid-lowering in lipid-associated tissue injuries has well been known, the potential benefits of statins beyond lipid-lowering are not well established [24]. Our previous study demonstrated that statins prevented inflammation induced by lipid in the kidney, which was not necessarily associated with its property inhibiting synthesis of cholesterol, but via directly acting on inflammasome. Here our data support a direct role of statins in suppressing ROS production induced by cholesterol by decreasing NOX2/NOX4 protein expression and improving mitochondrial dysfunction.

In cholesterol overload tubular cells, NOX4 is constitutively active producing hydrogen peroxide (H_2_O_2_) as the prevalent ROS detected, whereas other NOXs (NOX1, NOX2 and NOX5) present in the kidney generate superoxide radical anions as products [7]. The high expression of NOX4 would transfer electrons to reduce molecular oxygen to form O_2_^−^ which are considered as main producers of ROS. NOX4 is the crosslink between NAPDH oxidase and mitochondrial dysfunction and induce ROS production, as NOX4 exists in the outer mitochondrial membrane [25]. NOX4 upregulation decreased mitochondrial biogenesis and induced mitochondrial electron transport chain (mETC) deficiencies. In the present study, cholesterol markedly induced NOX4 protein expression and mitochondrial dysfunction, which was associated with ROS generation in the collecting duct cells. High-fat diet also induced NOX4 protein and gene expression, which presumably causing ROS in the kidney, leading to tubular injuries. Statins decreased protein/gene expression of NOX4 and improved mitochondrial function in vitro, which is supposed to be protective. Our previous study showed that statins ameliorated cholesterol-induced inflammation by promoting the degradation of NLRP3 inflammasome components in the kidney. Since NLRP3 inflammasome assembly could be triggered by ROS [14], ROS inhibition by statins is supposed to further attenuate NLRP3 activation. Therefore, the protective role of statins in lipid-induced kidney injuries is likely attributed to both inhibiting NLRP3 inflammasome activation and promoting degradation of NLRP3 components. Although the mechanism by which statins downregulates NOX4 expression was not examined, our data indeed support the conception that statins protect kidney injuries during obesity independent of its property of lowering cholesterol.

In conclusion, statins prevented ROS production induced by cholesterol in the kidney, likely through inhibiting NOX2 and NOX4 protein expression and improving mitochondrial function. Statins may be a therapeutic option in treating cholesterol-associated kidney diseases.

## Supplementary Information

**Additional file 1: Figure S1.** (A) Original western blot image for NOX2 in kidneys of 5/6Nx and high-fat diet rats with or without atorvastatin treatment. (B) Original western blot image for NOX4 in kidneys of 5/6Nx and high-fat diet rats with or without atorvastatin treatment. (C) Original western blot image for β-actin in kidneys of 5/6Nx and high-fat diet rats with or without atorvastatin treatment. **Figure 2.** (A) Original western blot image for NOX4 in cholesterol over loaded mpkCCD cells treatment with or without simvastatin. (B) Original western blot image for NOX2 in cholesterol over loaded mpkCCD cells treatment with or without simvastatin. (C) Original western blot image for Cleaved-caspase3 in cholesterol over loaded mpkCCD cells treatment with or without simvastatin. (D) Original western blot image for β-actin in cholesterol over loaded mpkCCD cells treatment with or without simvastatin.

## Data Availability

The datasets used and/or analysed during the current study available from the corresponding author on reasonable request.
