# Supplementary material for: Statins attenuate cholesterol-induced ROS via inhibiting NOX2/NOX4 and mitochondrial pathway in collecting ducts of the kidney
Source: BMC Nephrol. 2022 May 13;23:184. doi: 10.1186/s12882-022-02815-6 (PMC9102638; doi:10.1186/s12882-022-02815-6)
Supplement: Supplementary file 1 — Additional file 1: Figure S1. (A) Original western blot image for NOX2 in kidneys of 5/6Nx and high-fat diet rats with or without atorvastatin treatment. (B) Original western blot image for NOX4 in kidneys of 5/6Nx and high-fat diet rats with or without atorvastatin treatment. (C) Original western blot image for β-actin in kidneys of 5/6Nx and high-fat diet rats with or without atorvastatin treatment. Figure 2. (A) Original western blot image for NOX4 in cholesterol over loaded mpkCCD cells treatment with or without simvastatin. (B) Original western blot image for NOX2 in cholesterol over loaded mpkCCD cells treatment with or without simvastatin. (C) Original western blot image for Cleaved-caspase3 in cholesterol over loaded mpkCCD cells treatment with or without simvastatin. (D) Original western blot image for β-actin in cholesterol over loaded mpkCCD cells treatment with or without simvastatin. [file 12882_2022_2815_MOESM1_ESM.pptx]

## Slide 1
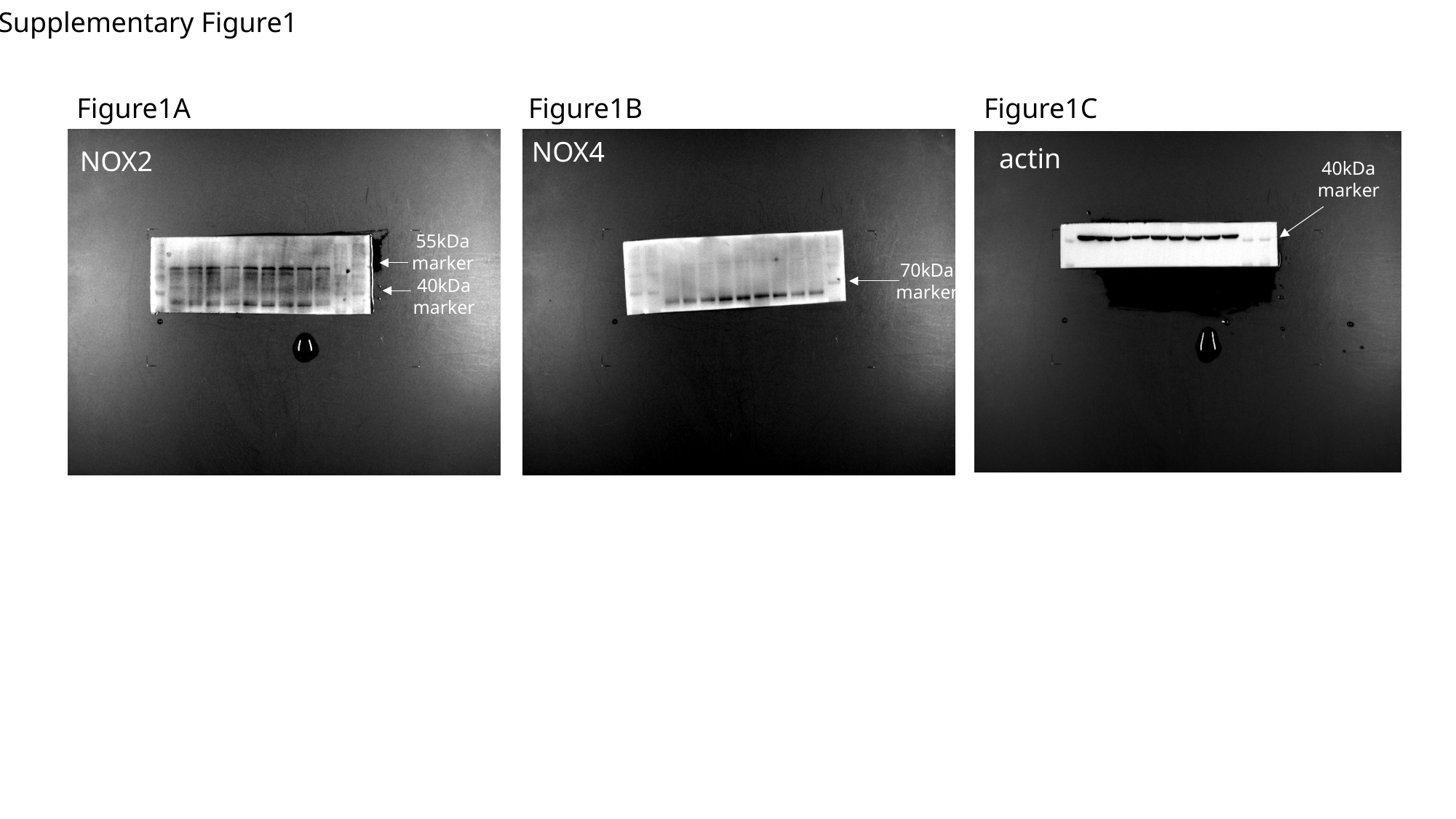

Supplementary Figure1
Figure1A
Figure1B
Figure1C
NOX4
actin
NOX2
40kDa
marker
55kDa
marker
70kDa
marker
40kDa
marker

## Slide 2
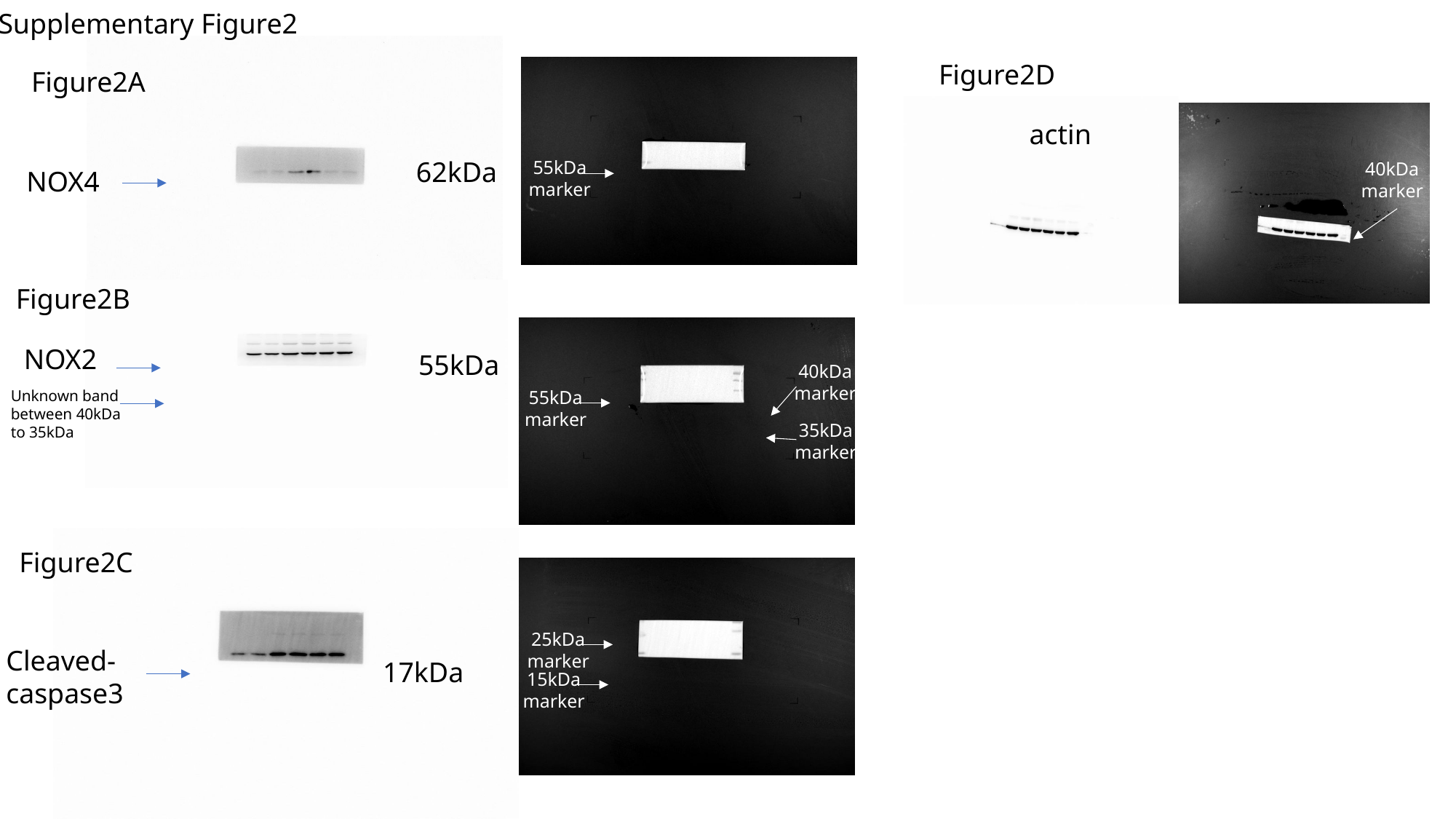

Supplementary Figure2
Figure2D
55kDa
marker
Figure2A
actin
62kDa
40kDa
marker
NOX4
Figure2B
NOX2
55kDa
40kDa
marker
55kDa
marker
Unknown band between 40kDa to 35kDa
35kDa
marker
Figure2C
25kDa
marker
Cleaved-
caspase3
17kDa
15kDa
marker
